# Functionalization of Biotinylated Polyethylene Glycol on Live Magnetotactic Bacteria Carriers for Improved Stealth Properties

**DOI:** 10.3390/biology10100993

**Published:** 2021-10-01

**Authors:** Richa Chaturvedi, Yumin Kang, Yunji Eom, Sri Ramulu Torati, CheolGi Kim

**Affiliations:** Department of Emerging Materials Science, DGIST, Daegu 42988, Korea; richachaturvedi@dgist.ac.kr (R.C.); rofl9000@dgist.ac.kr (Y.K.); yj0063@dgist.ac.kr (Y.E.)

**Keywords:** magnetotactic bacteria, biotin, polyethylene glycol, cytotoxicity, drug delivery, stealth property

## Abstract

**Simple Summary:**

The development of new approaches in the field of drug delivery systems is primarily based on increasing the accuracy and precision of the targeted site and improving the stability of the drug by preventing the phagocytosis process inside the body. Among many other methods used to fulfill the above-mentioned requirements, the use of magnetotactic bacteria (MTB) is proven to be a promising solution, as it is self-propelling in nature, and can also be controlled by an external magnetic field. For the present work, we developed an MTB/PEG–biotin complex by exploiting the process of covalent bond formation between bacteria and a biotin–PEG–NHS polymer. In addition to this, attachment efficacy and stability were also determined. In biological applications, cytotoxicity assay of THP-1 cells was performed, showing the MTB/PEG–biotin complex to be less harmful to the cells; meanwhile, to explore the stealth properties of the complex, we performed a cell association assay. With these results, we provide a significant contribution to the field of potential drug delivery system development.

**Abstract:**

The early removal of drug delivery agents before reaching the affected target remains an area of interest to researchers. Several magnetotactic bacteria (MTB) have been used as self-propelled drug delivery agents, and they can also be controlled by an external magnetic field. By attaching the PEG–biotin polymer, the bacteria are turned into a stealth material that can escape from the phagocytosis process and reach the area of interest with the drug load. In the study, we developed a potential drug carrier by attaching the PEG–biotin to the MTB-through-NHS crosslinker to form a MTB/PEG–biotin complex. The attachment stability, efficacy, and bacterial viability upon attachment of the PEG–biotin polymer were investigated. Biological applications were carried out using a cytotoxicity assay of THP-1 cells, and the results indicate that the MTB/PEG–biotin complex is less harmful to cell viability compared to MTB alone. Along with cytotoxicity, an assay for cell association was also evaluated to assess the complex as a potential stealth material. The development of these complexes focuses on an easy, time-saving, and stable technique of polymer attachment with the bacteria, without damaging the cell’s surface, so as to make it a strong and reliable delivery agent.

## 1. Introduction

In today’s modern era of disease diagnosis and treatment, various kinds of drug delivery and therapeutic agents—such as liposomes [1], micro/nanoparticles [2], and magnetic-based systems [3]—have become areas of interest for many researchers, as they promise to provide potential methods of treatment for many diseases. All of these kinds of therapeutics have been examined in clinical trials [4], as they use an active or passive targeting mechanism [5]. Although all of the important parameters—such as the cytotoxicity, shape, size, and surface chemistry—of such drug delivery agents have been investigated in vivo and in vitro [6], it remains a topic of concern to manage and examine their ability to escape phagocytic blood cells while also remaining at the area of interest for a longer duration. Among many other drug delivery agents, biotin [7] along with PEG [8] and NHS are of particular interest, as each of these materials plays an important role in drug delivery and bioconjugation processes. Biotin is known to be an important vitamin for the growth and survival of bacteria, and can also be utilized as a drug carrier for tumors, reducing the need for medications. Furthermore, biotin can release drugs in a highly acidic environment [9,10]. Along with these properties, biotin has an additional advantage of targeted drug delivery, as the blood has a relatively high pH compared to tumors and plaques; hence, a biotin-based carrier system would not release the drug before reaching the target site [10]. On the other hand, poly(ethylene glycol) (PEG)—an FDA approved material—acts as a biologically inert and non-immunogenic material, and PEG particles also possess neutral charge and extensive hydration in physiological solution; thus, PEG has been proven as a powerful stealth material [11,12]. PEG also increases the surface hydrophilicity and lessens the binding by phagocytic cells, where the materials with lower cell association show improved circulation time as well as better biodistribution [13].

Several approaches have been reported for targeted drug delivery towards tumors [10], plaques [14], or bacterial biofilms—which are hypoxic and acidic [15]—but the main drawback for these systems is their less studied and investigated pharmacokinetics and biodistribution [16]. Another major area of concern is that these targeted drug carriers have fixed limitations, as they are dependent on the host circulation system [15]. To overcome this issue, many biohybrid swimmers with active motile cells and made from artificial materials have been produced in recent studies [17]. To date, many drug delivery systems have been used, including contractive muscle cells [18], bacteria [19,20,21], and sperm cells [22], but their main problem is their pharmacokinetic response. To address this issue, *Magnetospirillum magneticum* AMB-1 magnetotactic bacteria (MTB) could be used as an alternative drug delivery agent [23]. These bacteria have all of the basic requisite components, namely, a sensory system that helps them to sense the oxic–anoxic interface (OAI)—a hotspot for their adaptation—a control system by which they navigate using the Earth’s magnetic field and, lastly, an actuation system that helps them in controlling their motility, which makes them an excellent drug delivery agent [24]. MTB is known to be a self-propelled organism, and its movement can be precisely controlled by an external magnetic field with the help of magnetosomes composed of superparamagnetic Fe_3_O_4_ or Fe_2_O_3_ nanoparticles [25]. Moreover, such an approach has been already shown to work properly in the hypoxic regions of tumors [20]. The swimming capabilities of any bacteria—including MTB—in low-Reynolds-number fluids are of great interest, as they can be applied in various microsystems [26]. Bacterial manipulation and actuation help the bacteria to carry cargos loaded with drugs to the affected area of the target, and this can be achieved by modifying the bacterial surface and the bacterial motion by applying an external magnetic field [15].

In this article, MTB was strategically attached to the PEG–biotin material to escape the phagocytes in a phagocytic assay before reaching the affected area, as PEG reduces cell association. The MTB/PEG–biotin complex was developed by the formation of a strong amide bond that relies on the concept of nucleophilic attack—releasing NHS in the process—and then thoroughly characterized for its structural properties, formation of strong bioconjugation, bacterial viability upon addition of PEG–biotin, cell viability, and cell association in THP-1 cells to demonstrate the reduced cell association of foreign particles with the phagocytes. This MTB/PEG–biotin complex could be used for the delivery of various drugs to the target site in an efficient way.

## 2. Materials and Method 

### 2.1. Reagents and Chemicals

The biotinylated PEG derivative with a terminal biotin group (Biotin-CONH-PEG-O-C_3_H_6_-CONHS) was purchased from Rapp Polymere GmbH (Tübingen, Germany). N-(3-dimethylaminopropyl)-N-ethyl carbodiimide hydrochloride (EDC), glutaraldehyde solution, fluorescein 5(6)-isothiocyanate (FITC), sodium nitrate (NaNO_3_), potassium dihydrogen phosphate (KH_2_PO_4_), L-ascorbic acid (C_6_H_8_O_6_), tartaric acid (C_4_H_6_O_6_), succinic acid (C_4_H_6_O_4_), sodium acetate (C_2_H_3_NaO_2_), iron(III) chloride (FeCl_3_), quinic acid (C_7_H_12_O_6_), and resazurin sodium salt were all purchased from Sigma-Aldrich (St. Louis, MO, USA). Phosphate-buffered saline (PBS), Roswell Park Memorial Institute (RPMI-1640) medium, fetal bovine serum (FBS), and penicillin/streptomycin were procured from Thermo Fisher Scientific (Waltham, MA, USA). Live/dead BacLight bacterial viability kits and live/dead viability/cytotoxicity kits were obtained from Invitrogen (Waltham, MA, USA). *Magnetospirillum magneticum* (ATCC 700264) bacteria, THP-1 cells (ATCC TIB-202), vitamin supplement (ATCC MD-VS), and trace mineral supplement (ATCC MD-TMS) were acquired from the American Type Culture Collection (Manassas, VA, USA). (2-(2-methoxy-4-nitrophenyl)-3-(4-nitrophenyl)-5-(2,4-disulfophenyl)-2H-tetrazolium monosodium salt (WST-8) (cell counting kit - 8, CCK-8) was purchased from Enzo Life Sciences, Inc. (New York, NY, USA). Methanol and ethanol were acquired from DaeJung Chemicals (Gyeonggi, Korea). High-purity (18 MΩ) water from the Milli-Q water system (Millipore, Bedford, MA, USA) was used for all of the experiments.

### 2.2. Magnetospirillum Magneticum AMB-1 Strain Culture Conditions

*Magnetospirillum magneticum* AMB-1 was cultured in ATCC-prescribed (1653) revised magnetic spirillum growth medium (MSGM) [27]. Briefly, the constituents of the MSGM medium were as follows (in grams per liter): 0.37 g of succinic acid, 0.05 g of sodium acetate, 0.37 g of tartaric acid, 0.035 g of ascorbic acid, 0.68 g of KH_2_PO_4_, 0.12 g of NaNO_3_, 10 mL of Wolfe’s vitamin solution, 5 mL of Wolfe’s mineral solution, and 2 mL of ferric quinate solution (dissolve 0.27 g of FeCl_3_ and 0.19 g of quinic acid in 100 mL of distilled water). The pH of the media was maintained at 6.75 with an aqueous sodium hydroxide solution. The microaerophilic environment was achieved by inoculating the bacteria in 25 mL of MSGM medium in a tightly screw-capped tube and incubating it at 30 °C for 5–6 days. Bacterial cells were harvested by centrifugation for conjugation with PEG–biotin to make a complex (MTB/PEG–biotin).

### 2.3. Attachment of PEG–Biotin to MTB

**Figure 1:**
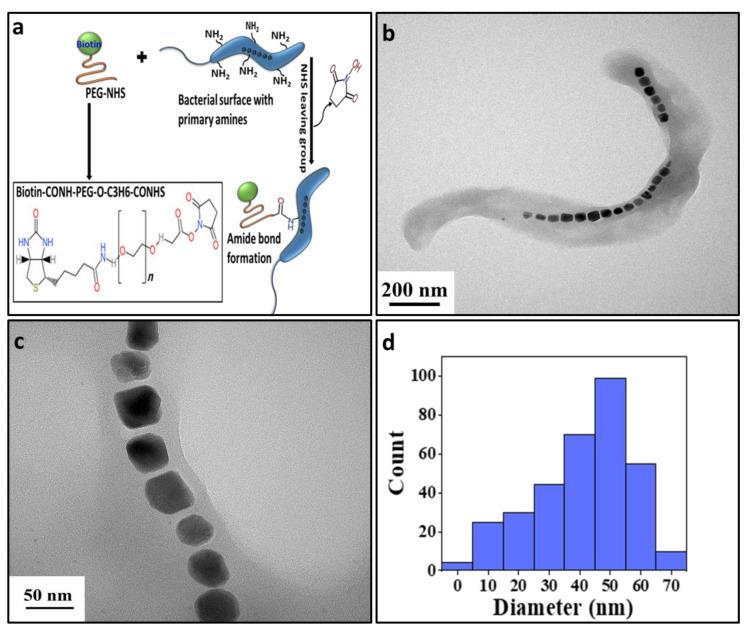
(**a**) Schematic representation of the loading of PEG–biotin on the bacterial surface; (**b**) TEM image showing the helical structure of MTB; (**c**) MTB with magnetosome particles; (**d**) histogram showing the size distribution of magnetosomes.

To successfully attach PEG–biotin to MTB, it is important to note that biotin–PEG–NHS, which is present in the Biotin–CONH–PEG–O–C_3_H_6_–CONHS reagent, reacts efficiently with the primary amine groups present on the surface of the bacteria (Figure 1a). Hence, this reagent was selected to form a complex with the MTB. The formation of the MTB/PEG–biotin complex was achieved via the following method: First, the biotin–PEG–NHS was dissolved in distilled water at 5 mg/mL concentration to make a stock solution of 10 mM. Likewise, different concentrations of stock solution (5, 10, 15, and 20 mM) were made by mixing a calculated amount of biotin–PEG–NHS in distilled water. In the next step, the conjugation was achieved by incubating 100 µL of each concentration of the stock solution of PEG–biotin with 3 × 10^7^ MTB cells, which were then kept in a rotator for 2 h at room temperature to permit amide bond formation. The covalent binding occurs when the biotin–PEG–NHS forms the amide bond, releasing the NHS by attacking a nucleophile, and subsequently forming a CONH bond. The MTB/PEG–biotin was separated from the unattached PEG–biotin with the help of a magnetic separator. Finally, the complex was rinsed with PBS three times, and then dispersed in PBS at pH 7.4 for further experiments.

### 2.4. Characterization of Magnetotactic Bacteria and Magnetosomes

The characterization of MTB and its magnetosomes was examined via transmission electron microscopy (TEM) and X-ray diffraction (XRD). The MTB was first grown in MSGM media for 6–7 days without any shaking, and then the bacteria were centrifuged at 7000 RPM for 20 min to separate them from their media, and then they were rinsed with PBS 3 times. The bacterial pellets were collected and incubated with 3% glutaraldehyde solution in PBS for 45 min at 4 °C for the fixation process, and then washed with water. A series of increasing ethanol concentrations (30%, 50%, 70%, 90%, and 100%) was used for the dehydration of the bacteria. In the process, the bacterial pellet was treated for 5 min in each solution (30–90%) and 10 min in pure ethanol. Then, the bacterial suspension was drop-cast on the TEM copper grid and air-dried prior to analysis. TEM images of bacteria and their magnetosomes were acquired using an FEI/Tecnai G2 F20 TWIN TMP microscope at 120 kV. The elemental composition was analyzed via energy-dispersive spectroscopy coupled with a TEM instrument. XRD patterns for the MTB to assess its structural properties were carried out using a Panalytical/Empyrean X-ray diffractometer. The intensity data were collected over 20–70° using step-scan mode (0.001°/s). For the XRD measurements, the samples were prepared by mixing the bacterial pellets with ethanol, and then drop-cast on the silicon wafer, followed by air-drying for 12 h prior to analysis.

### 2.5. Assessment of MTB/PEG–Biotin Complex Formation 

The formation of the MTB/PEG–biotin complex was examined via confocal laser scanning microscopy (CLSM), fluorescence-activated cell sorting (FACS), and field-emission scanning electron microscopy (FE-SEM). The fluorescent images of the MTB/PEG–biotin complex were acquired via CLSM (Carl Zeiss LSM 700). The PEG–biotin labeled with FITC was analyzed using up to four stable solid-state lasers (405/444, 488, 555, and 639 nm). The images were captured through a high numerical aperture (NA), 63 × 1.4 oil immersion objective, and imaging software (Zeiss ZEN LSM). The FITC-labeled MTB/PEG–biotin complex was assessed via fluorescence-activated cell sorting (FACS: BD Accuri C6, Biosciences, wavelength of 488 nm). Samples were taken in PBS, and measurements were carried out using forward scatter with fluorescence intensity plots for 10,000 events. Bare MTB was used as a control, and the results were analyzed using FlowJo software and suitable gates to generate histograms and density plots. The different thicknesses of PEG–biotin adhered to the bacteria surface were examined via FE-SEM (Hitachi/SU8230 FE-SEM). The MTB samples were made with different concentrations of PEG–biotin (10 and 20 mM) after 2 h of incubation. The bacterial sample for SEM analysis was made in the same way as for TEM analysis, but here it was drop-cast on a silicon wafer and then sputtered with platinum before imaging. The high-resolution SEM images were obtained at an accelerating voltage of 3–5 kV. 

### 2.6. Evaluation of Bacterial Viability with PEG–Biotin Attachment 

The viability of MTB/PEG–biotin was assessed using a live/dead BacLight bacterial viability kit. The kit contains two dyes: an SYTO that imparts green fluorescence to the live bacterial cells, and a propidium iodide that imparts red fluorescence to the dead cells. For the control, two kinds of bare MTB bacterial samples were prepared (live and dead), along with blank PEG–biotin. First, a concentration of 1 × 10^6^ MTB per well was incubated in the 96-well plates, in triplicate, and then PEG–biotin at different concentrations (5, 10, 15, and 20 mM) was added to the wells inoculated with MTB. The plates were then incubated for different time intervals (6, 12, and 24 h) before the addition of live/dead bacterial solution. The live/dead solution was made according to the manufacturer’s protocol; briefly, equal amounts of the two available dyes were mixed, and then made up to a 2X solution. After the addition of the solution, the 96-well plates were kept in the dark for 15 min before obtaining the readings. The fluorescence intensity of the samples was acquired using a microplate reader (BioTek Synergy H1). The bacterial viability data were analyzed by dividing the fluorescence intensity of live stained cells by that of dead stained cells. Next, the fluorescence images of the live/dead bacterial cells were obtained using an inverted fluorescence microscope (OLYMPUS IX83) after 6 h of incubation with the assay solution. 

#### 2.6.1. THP-1 Cell Culture

THP-1 (ATCC TIB-202) cells were cultured in RPMI 1640 medium, to which 10% fetal bovine serum (FBS) and 1% penicillin/streptomycin solution were added to prevent any kind of contamination. The cells were incubated at 37 °C in a humidified incubator with a supply of 5% CO_2_ for 2–3 days. The cells were harvested by centrifugation at 1200 RPM for 4 min, and then they were used for further experiments.

#### 2.6.2. CCK-8-Based Cell Viability Assay

The cytotoxicity effects of bare MTB and the MTB/PEG–biotin complex on THP-1 cells were evaluated via CCK-8 assay, which uses a WST-8 colorimetric assay. In the given assay, bare MTB and PEG–biotin were used as controls. First, THP-1 cells with a concentration of 2 × 10^6^ were seeded in the 96-well plates, in triplicate, and incubated in a suitable incubator. The THP-1 cell line was tested against bare MTB, PEG–biotin, and the MTB/PEG–biotin complex. The concentration of MTB was kept at 2 × 10^6^ MTB/well, with different concentrations of the PEG–biotin (5, 10, 15, and 20 mM). A CCK-8 solution was added to the plate, followed by incubation for 24 and 48 h. Briefly, after the decided incubation time, 10 µL of CCK-8 was added to each well, and again the samples were kept in the incubator for a further 2 h under 5% CO_2_ at 37 °C. The treated cells produced formazan dye, and its absorbance was measured with a microplate reader (BioTek Synergy H1) at a 450 nm wavelength. The cells alone without any MTB or the MTB/PEG–biotin complex were kept as controls. 

#### 2.6.3. THP-1 Cell Association

THP-1 cells taken at a concentration of 1 × 10^5^ cells were incubated with FITC-labeled bare MTB, PEG–biotin, and different concentrations of MTB/PEG–biotin complex (5, 10, 15, and 20 mM PEG–biotin) for 24 h at 37 °C in a 5% CO_2_ humidified atmosphere. After incubation, the cells were collected and rinsed thrice with PBS via centrifugation at 1200 RPM for 5 min. The collected cell pellets were resuspended in PBS and analyzed using a flow cytometer (BD Accuri C6). 

### 2.7. Statistical Analysis

Quantitative data are expressed as a mean ± standard deviation (SD). A two-way ANOVA (GraphPad Prism version 9.0 software) was used for the assessment of statistical significance. The number of independent variables is indicated in the figure legends. The *p*-values of 0.05 or less were set as the level of statistical significance for all of the tests.

## 3. Results and Discussion

### 3.1. Characterization of MTB

*Magnetospirillum magneticum* (strain AMB-1) is a Gram-negative helical-shaped MTB with an average diameter of 0.3–0.5 µm and length of 1–2 µm, as measured and analyzed using the TEM microscope (Figure 1b). The maximum size of the drug delivery system should not exceed 2 µm in diameter [28]; thus, the obtained size of the bacterium makes it a perfect drug delivery agent, as it is smaller than the microvasculature inside the body. The MTB possesses flagella for motility, and contains intracellular chains of magnetic nanoparticles called magnetosomes (Figure 1c) that guide the bacteria towards the preferred external magnetic field [29]. The magnetosomes in the bacteria were measured to be an average size of 50 nm (Figure 1d). The MTB produces different sizes of magnetosomes, as shown in Figure 1d, and the bacterial cell also makes chains of over 15 magnetosomes [30]. To confirm the presence of iron components, an elemental mapping analysis was carried out (Figure S1a), showing a high amount of iron distribution in the bacterial cell. An energy-dispersive X-ray (EDX) analysis (Figure S1b) was carried out to confirm the presence of constituent elements inside the bacteria along with the iron component. Figure S2 shows the XRD pattern of the magnetosomes that were present inside the bacterial cell. The peaks present at the 2θ range of 33.39° and 61.98° can be attributed to the rhombohedral (hexagonal) and crystalline structure of Fe_2_O_3_ particles. All of the peaks correspond to the pattern of hematite (JCPDS no.33-0664) [31]. The narrow and sharp peak indicates that the bacterial cells have produced highly crystalline hematite particles upon the usage of MSGM medium and incubation at 30 °C for 7–10 days [27].

### 3.2. Formation of MTB/PEG–Biotin Complex

**Figure 2:**
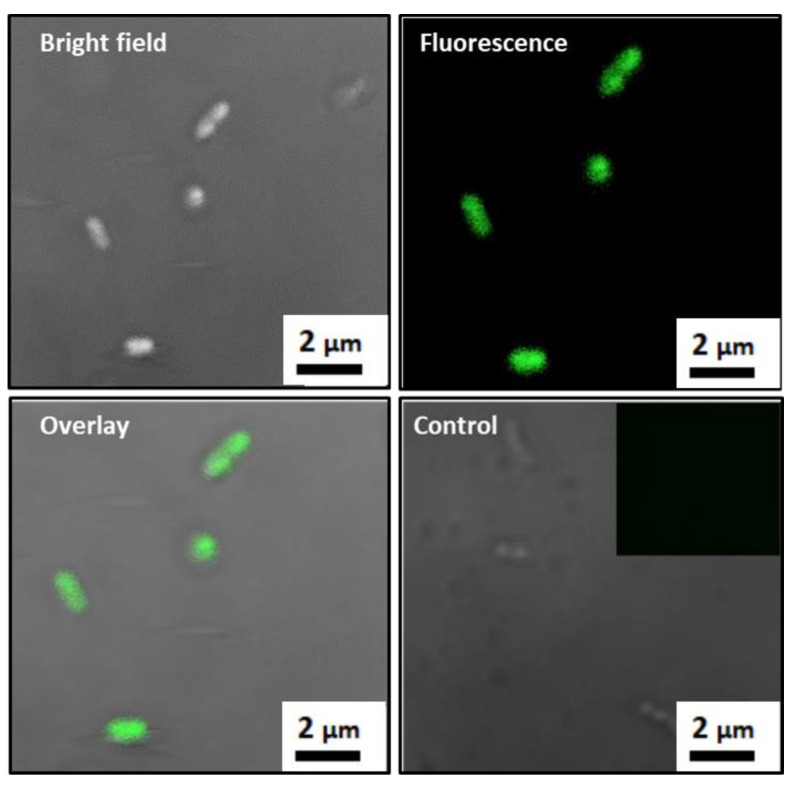
Confocal microscopy images of FITC-labeled MTB/PEG-biotin complexes. Photomicrographs show the bright field, fluorescence, and overlay confocal microscope images. Unlabeled bacteria are used as controls. Inset shows unlabeled bacteria (controls) that do not exhibit any fluorescence.

**Figure 3:**
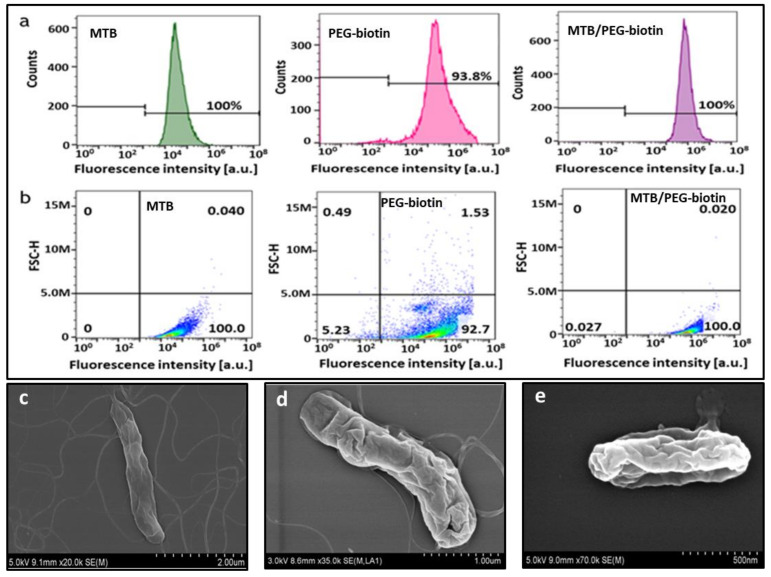
Characterization of the MTB/PEG–biotin complex. (**a**) FACS histograms and (**b**) density plots obtained from bare MTB, FITC-labeled PEG-biotin, and the MTB/PEG-biotin complex show that PEG–biotin is uniformly attached to MTB, and can be distinguished from bare MTB by fluorescence intensity. (**c**) FE-SEM images of bare MTB. MTB/PEG–biotin complex formation when the PEG–biotin concentration used was (**d**) 10 mM and (**e**) 20 mM.

The MTB/PEG–biotin complex was formed by incubating MTB bacterial cells with the PEG–biotin (Biotin–CONH–PEG–O–C₃H₆–CONHS) for two hours in a rotator. The biotin–PEG–NHS reagent took advantage of the amine groups that are abundantly found on the outer cell surface of Gram-negative MTB bacteria. Here, the NHS reacts with primary amines, such as the side-chains of lysines (K) or the amino-termini of polypeptides, and forms a strong amide bond via a nucleophilic attack and by releasing the NHS group [32]. To confirm the strong attachment of PEG–biotin to the bacterial cell surface, confocal laser scanning microscopy (CLSM) was carried out, where the localization of FITC-labeled PEG–biotin with the bacterial cells showed a good attachment of PEG–biotin to the surface of MTB which, in turn, assures a stable formation of the MTB/PEG–biotin complex (Figure 2). Here, the unlabeled bacteria were kept as controls; hence, the fluorescently labeled MTB/PEG–biotin complex can be distinguished clearly from the unlabeled bacteria, as the latter exhibit minimal or no fluorescence. The further confirmation of the successful complex formation was carried out via fluorescence-activated cell sorting (FACS). The histogram indicates that the sample with only bare bacteria exhibits a higher forward scattering (FSC) feature and a smaller amount of fluorescence compared to the fluorescently labeled PEG–biotin alone (Figure 3a). The bare bacteria were kept as negative controls. The MTB/PEG–biotin plot shows the integrated characteristics of PEG–biotin fluorescence intensity and the forward scattering feature of bare bacteria. The complex itself shows a wider distribution of fluorescence intensity compared to the bare bacteria, indicating a successful and strong bioconjugation between the bacteria and the PEG–biotin. The above-mentioned results were also proven by the density plot of the MTB/PEG–biotin complex (Figure 3b). From the MTB/PEG–biotin plot, it can be concluded that a considerable amount of PEG–biotin was attached to the bacteria via a strong and stable bond.

In addition to confocal microscopy and FACS analysis, FE-SEM was carried out to observe the morphological structure of the MTB/PEG–biotin complex. Differences between the SEM images of bare MTB bacterial cells and the MTB/PEG–biotin complex after vigorous washing confirmed a firm and strong binding of PEG–biotin to the bacterial cell membrane (Figure 3c–e). The thickness of the PEG–biotin adhered to the bacterial cell depends on its concentration. When a concentration of 3 × 10^7^ MTB cells was incubated with 100 µL of 10 mM PEG–biotin, it showed a thin layering of PEG-biotin around its cell membrane (Figure 3d), whereas a 100 µL of higher concentration of PEG–biotin (20 mM) displayed a thicker layer of PEG–biotin on the bacterial surface (Figure 3e). Hence, it can be concluded that the PEG–biotin is a versatile reagent used to biotinylate bacteria or proteins, making it a powerful tool for the formation of strong, stable, and irreversible amide bonds for in vivo and in vitro studies.

### 3.3. Assessing the Bacterial Viability for the MTB/PEG–Biotin Complex 

**Figure 4:**
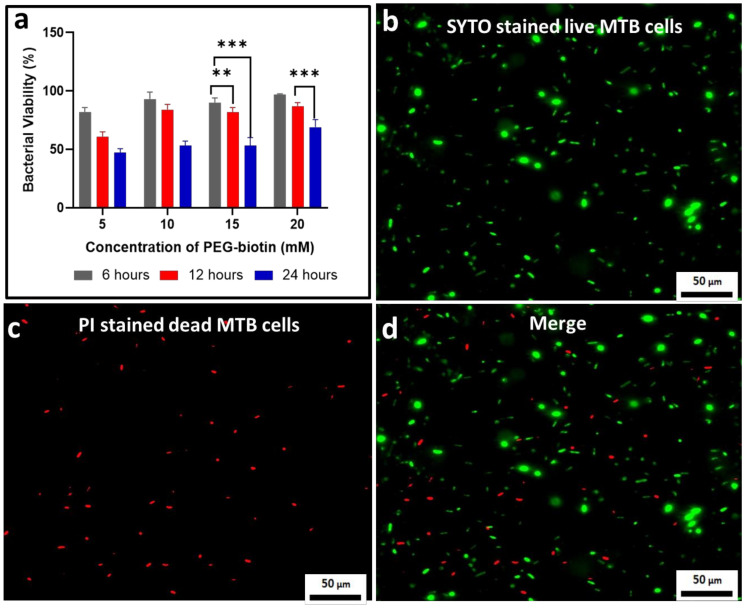
Assessment of bacterial viability. (**a**) Quantification of the percentage of bacterial viability where MTB was treated with different concentrations of PEG–biotin (5, 10, 15, and 20 mM) after different time intervals (6, 12, and 24 h). Results indicate that a higher concentration of PEG–biotin (20 mM) kept the bacteria alive even after long incubation times. Data are presented as mean ± SD, *n* = 3. Statistical significance (with *p* < 0.01 and *p* < 0.001) is indicated with ** and ***, respectively, for datasets that are significantly different between 6, 12, and 24 h. (**b**) Microscopic fluorescent live/dead images for live bacteria stained with SYTO stain; (**c**) bacterial cells stained with propidium iodide; (**d**) merged images for live/dead bacterial cells.

To determine the viability of MTB bacteria after the formation of the MTB/PEG–biotin complex, a bacterial viability assay was performed, and the results were obtained based on different concentrations of PEG–biotin (5, 10, 15, and 20 mM) and time intervals (6, 12, and 24 h). Figure 4a indicates that the bacteria incubated with 20 mM PEG–biotin show the highest viability percentage of 97%, whereas bacteria incubated with 5 mM PEG–biotin display a comparatively lower viability percentage of 82% after an incubation time of 6 h. Moreover, after the incubation of bacteria with 20 mM PEG–biotin for 24 h, they still show a remarkable percentage of 69% viability. The results are demonstrated by statistical analysis, represented by an asterisk. Hence, it can be concluded that the higher concentration of PEG–biotin keeps the bacteria viable even after a very long incubation time. This can be attributed to the fact that biotin increases the survival rate of bacteria, as it is an important vitamin for the growth and survival of bacteria [9]; thus, it makes it possible for MTB to survive for a longer time, and makes the MTB/PEG–biotin complex more stable. The viability of MTB treated with 20 mM PEG–biotin was estimated by using the live/dead bacterial viability kit. Figure 4b exhibits a high number of green-stained bacterial cells which, in turn, indicates the presence of live bacterial cells after incubation of 6 h; it also shows similarity with the live MTB cells, which are used as controls. On the other hand, the number of red-stained cells was small, indicating that the attachment of MTB to the 20 mM PEG–biotin (Figure 4c) did not greatly affect the viability of MTB bacterial cells when incubated with PEG–biotin for 6 h. Figure 4d shows merged images of live and dead bacteria. Therefore, it can be concluded that the MTB/PEG–biotin complex is safe for in vitro applications.

### 3.4. Biological Application of the MTB/PEG–Biotin Complex

**Figure 5:**
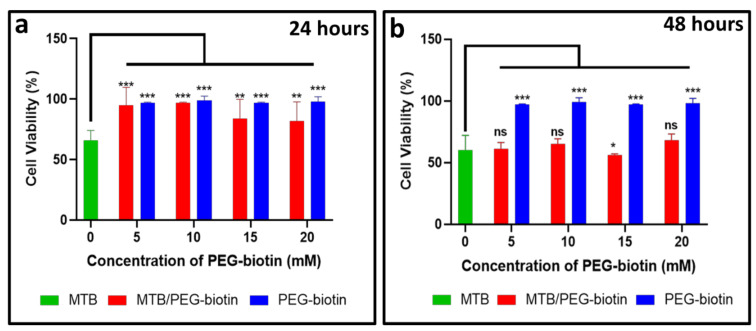
Cell viability assay. THP-1 cells were incubated for (**a**) 24 and (**b**) 48 h at 37 °C with different concentrations of PEG–biotin for the MTB/PEG–biotin complex, and compared to bare MTB and bare PEG–biotin, using the CCK-8 assay. The cell viability was found to be concentration and time-dependent. The results indicate that cells survive long after exposure to bare PEG–biotin and also the THP-1 cells survive long after incubation with MTB/PEG-biotin complex as compared to bare MTB. Statistical analysis was performed using two-way ANOVA. Data are presented as mean ± SD, *n* = 3. Significant difference was considered for * *p* < 0.05, ** *p* < 0.01, *** *p* < 0.001, and non-significant (ns) for *p* > 0.05.

**Figure 6:**
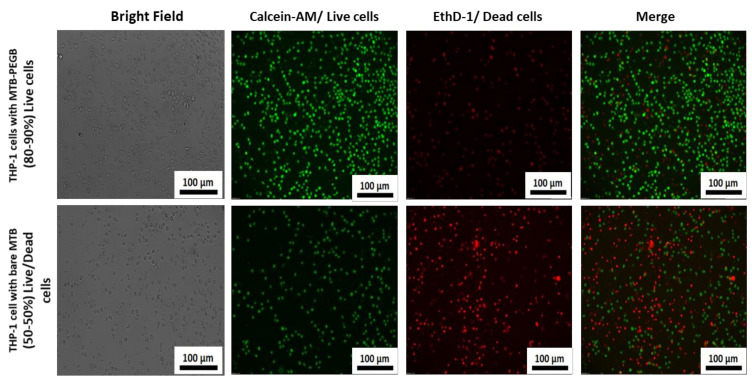
Bright-field and fluorescence microscopic images of live/dead assay: live/dead cell viability assay of THP-1 cells treated with bare MTB and the MTB/PEG–biotin complex. The cells were incubated for 24 h and then stained with a double-staining kit (calcein-AM and ethidium dimer-1). The live and dead cells exhibited green and red fluorescence, respectively. THP-1 cells treated with bare MTB showed 50% live cells, whereas cells treated with the MTB/PEG–biotin complex displayed 80–90% live cells.

Figure 5a,b display the cell viability of the THP-1 cell line for different concentrations of MTB/PEG–biotin complexes at different incubation times (24 and 48 h) using the cell counting kit-8, (CCK-8) assay. The viability of the tested cell line (THP-1) was time- and PEG–biotin-concentration-dependent. The THP-1 cell line is a widely used human monocytic cell line that has been commonly studied in phagocytosis assays. In this assay, the THP-1 cell line was tested against bare MTB, the MTB/PEG–biotin complex, and PEG–biotin alone. The concentration of MTB was kept at 2 × 10^6^ MTB/well for the present assay, and the different concentrations of PEG–biotin used were 5, 10, 15, and 20 mM. Most of the cytotoxicity was caused by the bare MTB, whereas THP-1 cells treated with the MTB/PEG–biotin complex exhibited no significant reduction in cell viability after 24 h of incubation. Moreover, the blank PEG–biotin at all concentrations did not cause any significant reduction in THP-1 cell viability, even after 48 h of incubation. The data were demonstrated using statistical analysis, where the effect of blank PEG–biotin on cells was almost non-significant, and the cells treated with the MTB/PEG–biotin complex exhibited significantly less decrease in their viability as compared to cells treated with bare MTB after 24 h of incubation. The significance is shown using an asterisk. Therefore, from this assay it can be concluded that the MTB/PEG–biotin complex caused less cytotoxicity (approximately 20-30%) to the tested THP-1 cell line compared to bare bacteria, signifying that the percentage of cell viability was improved by the attachment of PEG–biotin to MTB bacterial cells. In addition to the CCK-8 assay, the cell viability of THP-1 upon treatment with bare bacteria and MTB/PEG–biotin at a concentration of 20 mM was assessed using the live/dead viability/cytotoxicity kit. The THP-1 cells with bare bacteria and the MTB/PEG–biotin complex were incubated for 24 h. Figure 6 displays the microscopic images for the live/dead cell viability assay of THP-1 cells treated with bare MTB, and shows that almost 50% of cells were dead (red fluorescence) after 24 h of incubation, whereas the same THP-1 cells after treatment with the MTB/PEG–biotin complex for 24 h displayed almost 80–90% live cells (green fluorescence). Hence, the results obtained from the CCK-8 data show that the percentage of THP-1 viability was improved after treatment with the MTB/PEG–biotin complex, compared to bare MTB.

**Figure 7:**
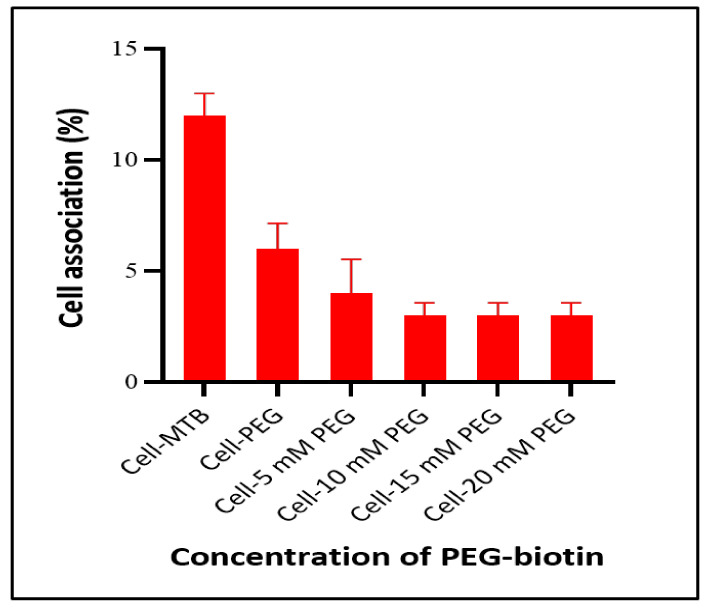
THP-1 cell association with different concentrations of PEG-biotin combined with MTB and bare MTB after 24 h of incubation at 37 °C. Results indicate that THP-1 cells incubated with bare MTB showed a very high cell association (12%), whereas cells treated with the MTB/PEG–biotin complex displayed relatively less cell association. Data were normalized to untreated cells. Error bars, mean ± SD, *n* = 4.

To explore the stealth properties of PEG–biotin, cell-association assessment of PEG–biotin with THP-1 cells was conducted with the help of fluorescence-activated cell sorting (FACS) after incubating the tested THP-1 cell line with bare MTB, bare PEG–biotin, and different concentrations of MTB/PEG–biotin (5, 10, 15, and 20 mM). As seen from Figure 7, less cell association was observed for the MTB/PEG–biotin complex compared to the bare MTB and PEG–biotin after 24 h of incubation with THP-1 cells. Moreover, MTB combined with a 5 mM concentration of PEG showed less than 5% cell association. The MTB/PEG–biotin complex with a 10 mM PEG concentration showed 3% cell association. Further increase in PEG concentration to 20 mM showed no significant decrease in cell association. THP-1 cells conjugated with the bare bacteria showed the highest percentage of cell association. The low percentage of THP-1 cell association with MTB/PEG–biotin indicates that magnetotactic bacteria conjugated with biocompatible PEG–biotin reduces association and uptake by phagocytes. This can be attributed to the fact that the higher molecular weight of PEG results in a thicker protective layer [33], and PEG is a biologically inert and non-immunogenic material [34]. Furthermore, the PEG present in the reagent provides hydration to the biotinylated object, helping to prevent aggregation of MTB stored in the solution [35]. The spacer arm reduces the steric hindrance while binding with any drug [36]. This can be an important finding, as many drug delivery agents are deficient in escaping phagocytosis, and are thrown out of the body quite early, without even working properly on the target area. Hence, the PEG provides a much-needed stealth property to the drug delivery agent.

## 4. Conclusions

We developed a therapeutic drug delivery agent by combining a polymeric PEG–biotin linker with MTB via the coupling of PEG–biotin to the naturally existing reactive amine groups that are present abundantly on the bacterial surface by the formation of a strong amide bond. The viability of the MTB does not change upon the conjugation with PEG–biotin, and it can be used for long-duration experiments. The attachment of biocompatible PEG–biotin improves the cellular viability of the complex, and also reduces the cell association and uptake by phagocytes. The developed agent is appropriate for systemic biocompatibility and cytotoxicity, and also shows stealth properties, escaping association by phagocytes. This complex shows the potential for application in improved biodistribution, medical imaging, and drug delivery and release in acidic environments of tumors or atherosclerotic plaques, as well as the ability to remain for a longer time at the target areas. Our developed complex can potentially overcome the challenges of phagocytosis and early removal of foreign objects by our immune system which, in turn, can give the drug carrier enough time to distribute the drug to the affected area. However, future studies will investigate whether the PEG–biotin detaches from the bacteria in an in vivo environment, or manages to remain intact as it is. Furthermore, the role of biotin can be examined as a drug delivery agent, as in some studies it has been shown that biotin can release drugs in an acidic environment. Hence, the proposed MTB/PEG–biotin complex can serve as a promising drug delivery agent, as it has stealth properties that help the system to work properly in an in vitro model.

## Data Availability

The data is contained within the article and supplementary material.

## Supplementary Materials

The following are available online at https://www.mdpi.com/article/10.3390/biology10100993/s1, Figure S1: (a) Elemental mapping analysis and (b) energy dispersive X-ray (EDX) spectrum of magnetotactic bacteria, Figure S2: XRD patterns of iron oxide (Fe2O3) obtained from the magnetosomes of magnetotactic bacteria.
