# Supplementary material for: Functionalization of Biotinylated Polyethylene Glycol on Live Magnetotactic Bacteria Carriers for Improved Stealth Properties
Source: Biology (Basel). 2021 Oct 1;10(10):993. doi: 10.3390/biology10100993 (PMC8533374; doi:10.3390/biology10100993)
Supplement: Supplementary file 1 [file biology-10-00993-s001.zip › biology-1372590-supplementary.pdf]

# Supplementary Information

# Functionalization of the biotinylated polyethylene glycol on live magnetotactic bacteria carrier for improved stealth property


Richa Chaturvedi, Yumin Kang, Yunji Eom, Sri Ramulu Torati*, CheolGi Kim*

*Department of Emerging Materials Science, DGIST, Daegu, 42988, Republic of Korea.*

*Corresponding authors: E-mail: srtorati@dgist.ac.kr (SR Torati) cgkim@dgist.ac.kr (CheolGi Kim), Tel: +82-53-785-6516, Fax: +82-53-785-6509

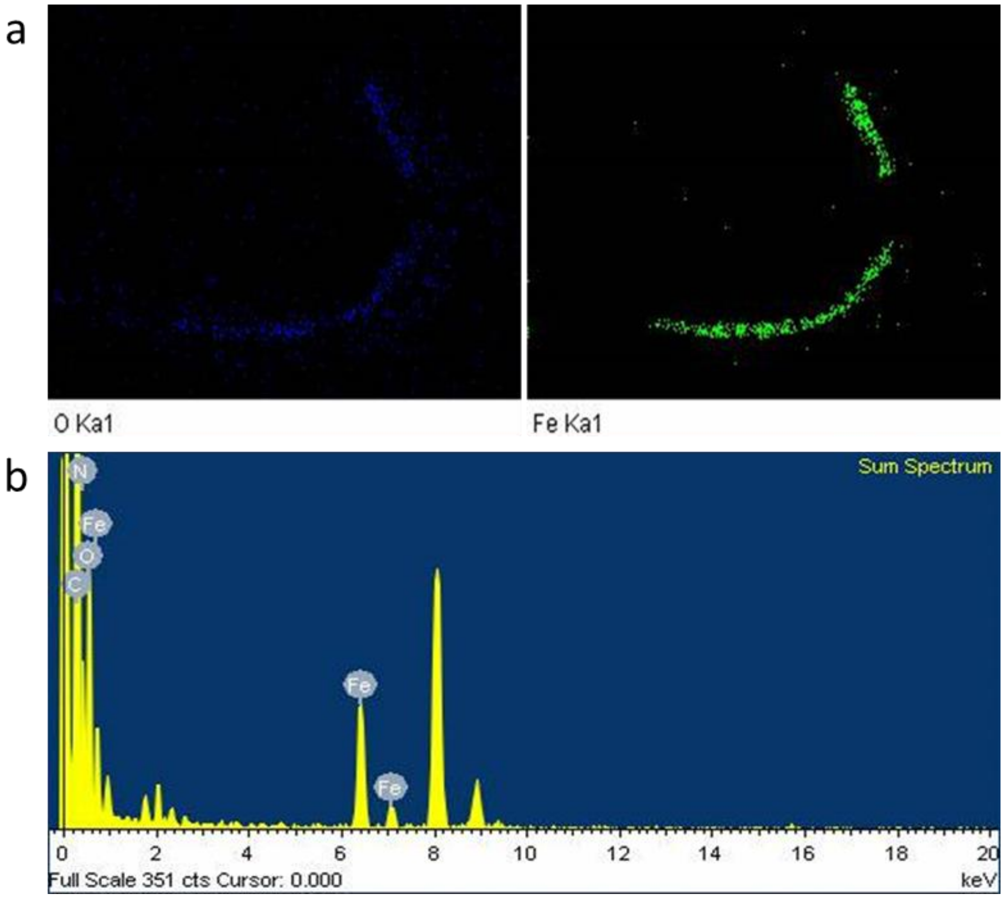


**Figure S1:** (a) Elemental mapping analysis and (b) energy dispersive X-ray (EDX) spectrum of magnetotactic bacteria

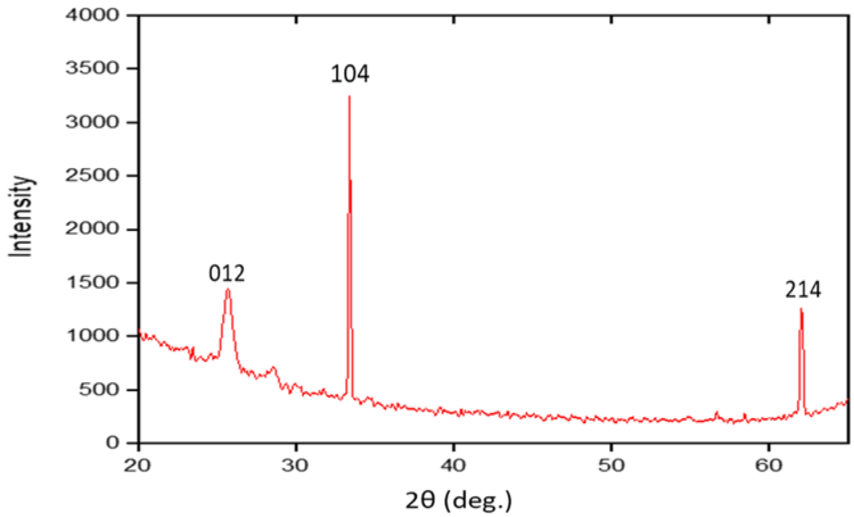


**Figure S2.** XRD patterns of iron oxide ($Fe_2O_3$) obtained from the magnetosomes of magnetotactic bacteria
